# Unraveling the Association Between Myocardial Infarction of Nonobstructive Coronary Arteries and Antiphospholipid Syndrome

**DOI:** 10.7759/cureus.17002

**Published:** 2021-08-08

**Authors:** Vishal Ramjas, Arpit Jain, Rholter Dave M Lee, Fioni Fioni, Nouran Tawfik, Osama Sandhu, Pousette Hamid

**Affiliations:** 1 Medicine, California Institute of Behavioral Neurosciences & Psychology, Fairfield, USA; 2 Neurology, California Institute of Behavioral Neurosciences & Psychology, Fairfield, USA

**Keywords:** myocardial infarction with nonobstructive coronary arteries, myocardial infarction with normal coronary arteries, minoca, coronary microvasculopathy, thrombosis, cardiovascular, intracardiac thrombosis, antiphospholipid syndrome, anti‐β2gpi, antiphospholipid antibodies

## Abstract

The term "myocardial infarction with nonobstructive coronary arteries (MINOCA)" refers to a condition characterized by clinical signs and symptoms consistent with acute myocardial infarction (AMI) (as defined by the third universal definition of infarction) and coronary arteries that are angiographically normal or nearly normal. A prominent source of morbidity and mortality in patients with antiphospholipid syndrome (APS) is thrombotic events. To evaluate whether there is a relation between APS and MINOCA in this research, we did an extensive assessment of the existing research in this field. According to the data, APS was associated with microvascular thrombosis, aberrant lipid metabolism, hypertension, and abnormalities of the coagulation cascade, among other conditions. Based on the available data, we discovered evidence that suggests a relationship between MINOCA and APS patients. It is vital to raise awareness of this concern among the general public. Also required is the development and implementation of diagnostic and targeted treatment guidelines for patients with APS and MINOCA.

## Introduction and background

"It's not the hole in the doughnut where the action is. It's the doughnut itself."

- Steven E. Nissen, MD [1].

Acute myocardial infarction (AMI) with nonobstructive coronary artery disease accounts for five to six percent of all AMI patients undergoing coronary angiography [2]. These cases are an interesting subgroup called myocardial infarction (MI) of nonobstructive coronary arteries (MINOCA) [3]. In previous researches, obstructive coronary artery disease (CAD) has been identified as epicardial artery stenosis of ≥50% on coronary angiography, whereas obstruction of <50% is needed for the diagnosis of MINOCA [3,4]. MINOCA's potential underlying mechanisms include cardiac spasm, coronary microvascular dysfunction, Takotsubo cardiomyopathy, and myocardial diseases such as myopericarditis and thrombophilia states [5].

Antiphospholipid syndrome (APS) is a chronic inflammatory disease of arterial and venous thrombosis that frequently correlates with elevated titers of antiphospholipid (APL) antibodies such as anti-cardiolipin (anti-ACL), lupus anticoagulant (LAC), and anti-β2-glycoprotein I (anti-β2GPI) acting on the phospholipids of the cell membrane [6,7]. The insult to coronary arteries in patients with APS is hypothesized to be directly related to accelerated atherosclerosis caused by an underlying autoimmune disorder like systemic lupus erythematosus in patients with APS [8]. However, in the absence of typical cardiovascular risk factors or atherosclerosis, ischemic arterial attacks in patients with APS may occur - without any underlying systemic disorders [8]. APL antibodies can induce thrombosis in any vascular bed, even coronary artery circulation, unlike congenital thrombophilia which is primarily associated with venous thrombosis [9].

The incidence of APS leading to AMI is rare, with a general prevalence of 5.5% [10]. It is even more uncommon when APS is the primary pathology [10]. The prevalence of AMI in young adults with APS is less than 2.8% [11]. Due to accelerated atherosclerosis in such patients, cardiovascular disease (CVD) is the leading cause of death, often progressing more rapidly than the general population [12]. Clinically silent myocardial ischemia, elevated pulmonary pressure, and coronary atherosclerosis are present in a large proportion of APS patients [13,14]. When comparing causes of death, MI was the leading cause, contributing to 19% of deaths in patients with APS over a five-year follow-up period [15].

The correlation between APS and non-thrombotic AMI is not always apparent [5]. However, it remains a clinical challenge to prevent misdiagnosing young people with APS as having a MI [8]. Currently, very few studies have attempted to delineate the mechanism describing the association between MINOCA and APS, hindering the process of developing necessary guidelines for patient diagnosis and therapy [5]. Studies exploring the association between APS and MINOCA morbidity and mortality are in their infancy phase. In this review, we will utilize the PubMed and Google Scholar databases to search the available literature and to study the association of APS and MINOCA development.

## Review

APS and cardiovascular events

APS is characterized as a condition of hypercoagulability secondary to the presence of APL antibodies, a group of autoantibodies directed toward plasma proteins that interact with membrane phospholipids [11,16]. Additionally, non-inflammatory myocardial microvasculopathy occurs in patients with APS who do not have any clinical or immunological evidence of systemic lupus erythematosus or any other disease [17,18].

Myocardial infarction with nonobstructive coronary arteries (MINOCA) is a condition characterized by clinical signs of AMI (according to the third universal concept of infarction) and angiographically intact or almost normal coronary arteries. The cut-off commonly used in the literature to describe stenosis as nonobstructive is when the lumen is less than 50% obstructed [4,19].

**Figure 1:**
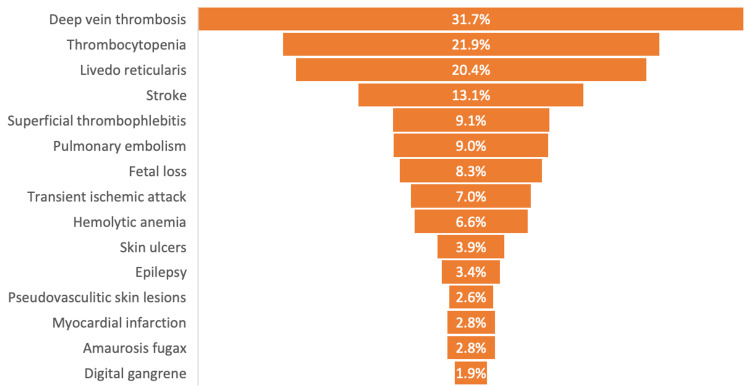
Clinical manifestations of patients with antiphospholipid syndrome Sources: References [17] and [18].

While venous thromboembolism is the most common pathological manifestation in APS patients, a proportion of patients experience thrombosis in arteries (acute coronary syndrome, stroke, TIA) [16]. In addition to cerebrovascular symptoms, cardiac events often constitute a significant cause of morbidity and mortality in APS patients. These involve a broad range of clinical conditions and symptoms of coronary or non-coronary organs. In 2.8% of APS cases, patients present with AMI (Figure 1) [16,20,21].

In 2019, Tamis-Holland et al. reported that MINOCA occurs when thrombosis or embolism involves coronary microvasculature or partial lysis of epicardial coronary thrombus resulting in nonobstructive angiographic disease. This can happen with or without the presence of a hypercoagulable state. They discovered that 7.5% of patients with coronary embolism have APS [2,3,22].

While typical MI with occluded coronary arteries in APS patients is well described, there are also many reports of anginal chest pain and MI with a nonobstructive coronary artery in patients with APS [23]. In 2017, Nazir et al. searched the medical literature and found 40 published cases of AMI secondary to APS. Patients were younger than average AMI patients (41.10 ± 13.61 years), with 45% being female. In 18/40 cases (45%), AMI presented as ST-segment elevation. They found this in 75% of the cases presented with coronary arteries that were either normal or thrombotic. Three died while in the hospital, and six had a recurrence of MI within three months of being admitted [24].

In 2019, Gandhi et al. conducted a study where cardiac catheterization was performed in 40 patients with AMI out of 575 patients with positive APL antibodies. Of these, eight patients were diagnosed with MINOCA. Their analysis showed that vasospasm might have played a role in two out of the eight patients, which was not previously related to APS. The etiology was unknown for the remaining six patients [5]. In 2004, Zavaleta et al. reported that after a five-year follow-up study, six of the 24 patients with AMI had coronary angiograms that revealed nonobstructive coronary arteries, implying the risk of embolic incidents [25].

We conducted a search of medical literature published between 2003 and 2019 to investigate if APS was associated with cardiovascular events. Collectively, these results established that patients with APS developed coronary artery ischemia with nonobstructive coronary arteries based on molecular imaging, echocardiography, or pathology records. Four out of eight articles were limited to their small sample size. A few articles hypothesized the process by which the heart is injured in MI. Due to the paucity of data, further studies are necessary.

Risk factors

Thrombotic events are a significant source of morbidity and mortality in APS. Apart from the APL antibodies, traditional risk factors for CVD may coexist in these patients, increasing their risk of developing thrombosis [6,26].

Traditional risk factors for atherosclerosis include hypertension, diabetes, obesity, dyslipidemia, smoking, and a sedentary lifestyle. These comorbidities can affect inflammatory pathways and lipid metabolism, leading to vascular injury, thus triggering and propagating atherosclerotic plaque formation. Similar mechanisms are involved in the vascular activity in the APS patients [27].

According to de Souza et al., hypertension is the typical cardiovascular risk factor most closely associated with APS's arterial thrombotic phase. Their research looked at the prevalence of typical risk factors for CAD and other causes in 38 patients with predominant APS and 30 healthy controls to see if there was a connection to arterial thrombosis. The study population had a higher overall number of risk factors, with hypertension being the only one independently associated with arterial thrombosis. Furthermore, patients had higher low-density lipoprotein (LDL) levels and triglycerides and lower high-density lipoprotein (HDL) levels than those in the APS group when compared to the control group [6,28].

In 2017, Radin et al. demonstrated the therapeutic usefulness of the modified Global Antiphospholipid Syndrome Score (aGAPSS) for risk stratification of AMI in a group of young patients with APS. Higher aGAPSS values were found in patients with AMI. Significantly higher aGAPSS levels were also seen in patients with acute coronary syndrome relative to cerebrovascular arterial thrombotic events. Risk stratification of APS patients under the age of 50 for the possibility of experiencing acute coronary thrombotic events may direct preventative pharmacological care for high-risk patients [29].

We conducted a review of studies published between 2006 and 2019 that examined the association between APS and CVD risk factors and the importance of the modified aGAPSS for risk stratification of AMI in a group of young patients with APS. Koniari et al. found that accelerated atherosclerosis raises the likelihood of coronary heart disease; the etiology seems to be more linked to inflammatory and immunopathologic causes than conventional Framingham cardiovascular risk factors [30]. These studies established that APS patients with elevated blood pressure, altered lipid metabolism, and coronary atherosclerosis have an increased risk of developing cardiovascular complications. As a result of the plurality of recent research, it can be inferred that APS is associated with CVD risk factors.

Pathophysiology

The pathophysiology of MINOCA is complex and multifactorial [31]. The causes of MINOCA are many and can be categorized as epicardial (unstable plaque not visible on angiography, epicardial spasm, and coronary dissection) or microvascular. The latter is further subdivided into intrinsic (microvascular spasm, Takotsubo syndrome, and coronary embolization) and extrinsic (myocarditis and other conditions) [19].

**Figure 2:**
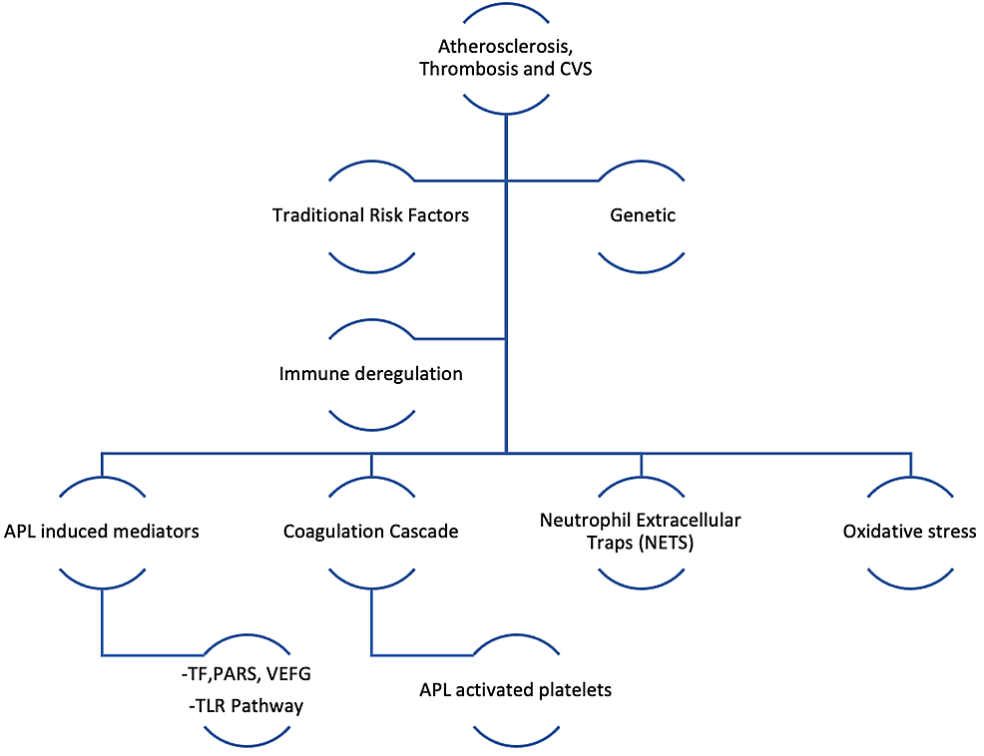
Mechanisms of atherosclerosis, thrombosis, and cardiovascular disease in APS APS, antiphospholipid syndrome; CVS, cerebral venous sinus; TF, tissue factor; PARs, protease-activated receptors; VEGF, vascular endothelial growth factor; APL, antiphospholipid.

Coronary thrombosis may be caused by inherited or acquired thrombotic diseases, whereas coronary emboli can be caused by coronary or systemic arterial thrombi. Thrombophilia screening studies in patients with MINOCA associated with hereditary thrombophilia include Factor V Leiden thrombophilia and protein S and C deficiency. Additionally, acquired thrombophilia disorders include APS and myeloproliferative disorders (Figure 2) [32].

**Figure 3:**
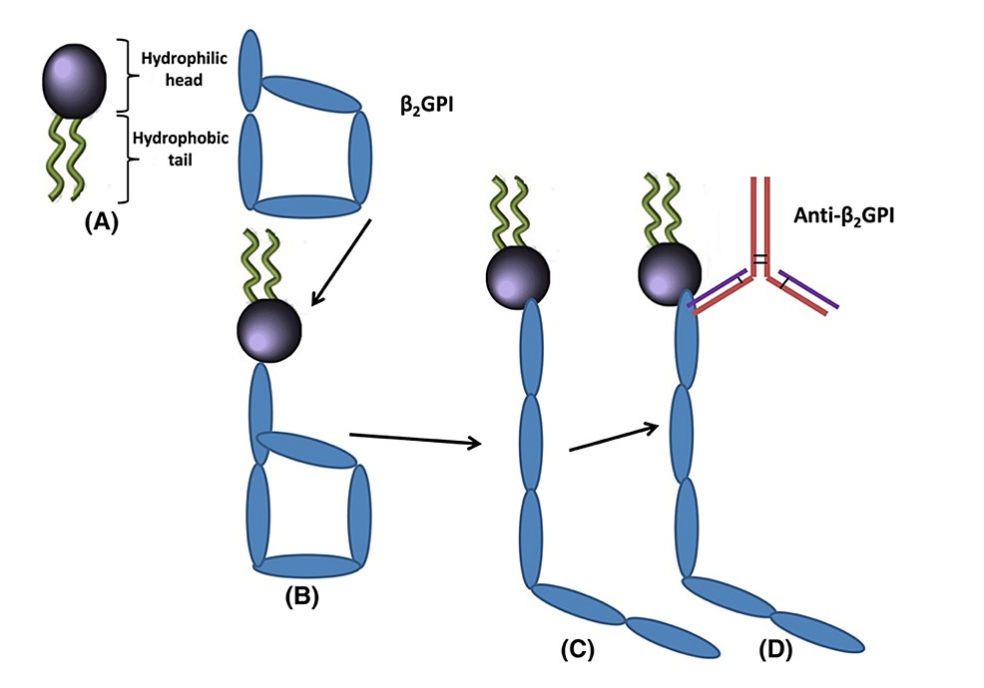
(A-D) Schematic of the folded (circular) and unfolded conformations of beta2 glycoprotein I (2GPI) and subsequent binding to anti-2GPI antibodies Source: Reference [32].

APL antibodies include a diverse array of autoantibodies that include lupus anticoagulant (LA), immunoglobulin (Ig)G, IgM anti-cardiolipin antibodies (ACL), and anti-2-glycoprotein I (anti2GPI) antibodies. Anti2GPI has the strongest correlation with the pathogenicity of the various plasma proteins antibody ligands in APS (Figure 3) [33-35]. Platelets, monocytes, and endothelial cells are stimulated, triggering the expression of tissue factor (TF, the primary inducer of coagulation in vivo), protease-activated receptors (PARs), and proinflammatory cytokines, a mechanism that ultimately results in thrombus [36,37].

In addition, APLs foster oxidative and mitochondrial dysfunction, which induces an inflammatory profile with increased levels of many cytokines, chemokines, and endothelial damage promoters [36,38].

In 2016, Pérez-Sánchez et al. established a correlation between microRNAs and CVD in APS. They concluded that miRNAs were significantly decreased in neutrophils infected with APL-IgG or anti-dsDNA-IgG relative to those treated with synthetic human IgG, while miR-155 and miR-146a tended to be increased in monocytes. Numerous miRNAs were shown to be compatible with molecules associated with oxidative stress, inflammation, and thrombosis and were associated with the presence of atheroma plaques (identified by increased carotid intima-media thickness [CIMT] in Echo-Doppler analyses) and thrombotic cases [39,40].

**Figure 4:**
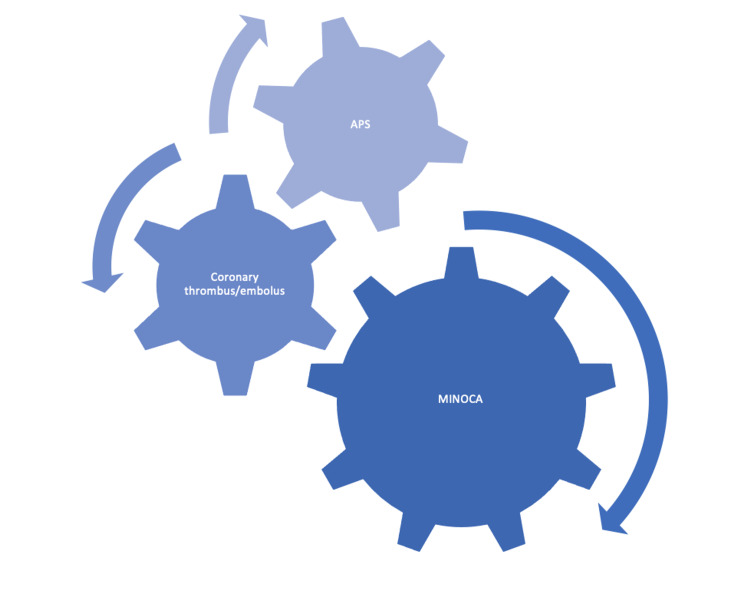
Microvascular causes of MINOCA and association with APS MINOCA, myocardial infarction with nonobstructive coronary arteries; APS, antiphospholipid syndrome

In 2015, Yalavarthi et al. demonstrated that APL might stimulate neutrophils and cause the release of NETs, a form of neutrophil cell death armed that results in the externalization of granular and nucleolar proteins that trigger pathologic platelet aggregation, implying that these circulating NETs can lead to thrombotic events (Figure 4) [41,42]. In 2014, Benhamou et al. reported that patients with primary arterial APS that do not have a history of atherosclerosis show endothelial deterioration and intrinsic arterial modifications and activation of the TLR2 and TLR4 signaling pathways, which is consistent with a systemic inflammatory, prooxidative, and prothrombotic condition [43,44].

APS and silent ischemia

In 2019, Cranley et al. reported a 51-year-old woman with known primary APS diagnosed with inferior ST-segment elevation and a normal emergency coronary angiography. However, MRI with late gadolinium enhancement confirmed subendocardial changes led to a preliminary diagnosis of MINOCA. This emphasizes the role of cardiovascular magnetic resonance (CMR) in detecting ischemic heart lesions early and motivating further cardiac investigation and the early initiation of cardioprotective therapy [45].

In 2016, Padjas et al. concluded that single-photon emission computerized tomography (SPECT) demonstrates myocardial perfusion defects with coronary calcifications in approximately one-sixth of them in comparatively young APS patients. Thus, a significant proportion of patients with APS have clinically silent myocardial ischemia and coronary atherosclerosis, both of which are causally linked to the presence of APL antibodies. This raises the question of how many young patients are at risk [13].

Riga et al. described a 16-year-old girl with MI whose coronary-computer tomography revealed no coronary defects or obstruction but had elevated three APL antibody levels. Myocardial magnetic resonance imaging revealed transmural necrosis with microvascular obstruction in the left ventricle's inferobasal section, indicating a microvascular myocardial infarction [46].

In 2012, Onea et al. discovered a small apical region of subendocardial delayed contrast enhancement, indicative of ischemic damage, in a 57-year-old patient two weeks later during a CMR test, implying that systemic APS-related coagulopathy played a significant role [15,47].

In 1992, Kattwinkel et al. reported a case of cardiac necrosis due to myocardial microvasculopathy in the absence of vasculitis in a patient with primary APS. This case showed undeniably that non-inflammatory myocardial microvasculopathy could arise in the primary APS in the absence of clinical or immunologic evidence of systemic lupus erythematosus or another disease method [17]. Pervez et al. found that a coronary angiogram revealed acute in-situ thrombosis in a 27-year-old patient but no indication of underlying atherosclerotic CAD. They demonstrated that AMI is managed similarly in patients with APS in the general population during the acute period [7].

In our review of papers published between 1992 and 2019 to ascertain if APL would result in MI in patients with nonobstructive coronary arteries, seven studies demonstrated that APL could result in thrombotic complications and MI in patients who lack conventional risk factors and have normal coronary arteries. Abid et al. conducted a systematic review and reported that the coronary arteries of 21 patients diagnosed with a Q-wave MI were shown to be normal. Coagulation disorders, including activated protein C (APC) resistance, protein C deficiency, and APL antibody syndrome, were identified in four out of 12 patients [48]. As a result, it can be concluded that APL is associated with CVD and can result in silent ischemia in patients with normal coronary arteries. These studies, however, were constrained by their restricted sample sizes, and thus additional larger studies are needed.

Limitations

Some publications in this research were more than 10 years old. Additionally, it lacked large-scale epidemiological investigations; the bulk of investigations had a small sample size.

## Conclusions

The purpose of this paper was to conduct a review of the current literature to determine the relationship between APS and MINOCA. As a result, we concluded that APS was linked with MINOCA and ischemic CAD. There is scant evidence opposing this relationship. Other autoimmune disorders, such as systemic lupus erythematosus, in people with MINOCA and APS, might be a confounding factor. In summary, persons with APS and CVD risk factors such as hypertension and dyslipidemia are at the greatest risk of developing MINOCA.

Additionally, further research is necessary to define APS's specific pathogenicity and compile an accurate census of persons who may be at risk. Surveillance of APS patients and raising public awareness should also be encouraged. Furthermore systematic and meta-analyses are required to establish essential criteria for diagnosis and treatment for patients with MINOCA and APS.
